# Mental Health Professionals’ Perspectives on Digital Remote Monitoring in Services for People with Psychosis

**DOI:** 10.1093/schbul/sbaf043

**Published:** 2025-05-07

**Authors:** Hannah Ball, Emily Eisner, John Ainsworth, Eloise Bagg, Louise Beattie, Matteo Cella, Natalie Chalmers, Sybil Clifford, Richard J Drake, Sophie Faulkner, Kathryn Greenwood, Andrew Gumley, Gillian Haddock, Kimberley M Kendall, Alex Kenny, Jane Lees, Shôn Lewis, Laura Maclean, Jennifer Nicholas, Kathryn O’Hare, Anuoluwapo Oluwatayo, Sandapa Punchihewa, Cara Richardson, Leonie Richardson, Matthias Schwannauer, Joseph Sherborne, Rebecca Turner, Evelin Vogel, James Walters, Alice Warner, Paul Wilson, Til Wykes, Uzma Zahid, Xiaolong Zhang, Sandra Bucci

**Affiliations:** Division of Psychology and Mental Health, Faculty of Biology, Medicine and Health, Manchester Academic Health Sciences, The University of Manchester, School of Health Sciences, Manchester, United Kingdom; Greater Manchester Mental Health NHS Foundation Trust, Manchester, United Kingdom; Division of Psychology and Mental Health, Faculty of Biology, Medicine and Health, Manchester Academic Health Sciences, The University of Manchester, School of Health Sciences, Manchester, United Kingdom; Greater Manchester Mental Health NHS Foundation Trust, Manchester, United Kingdom; Division of Informatics, Imaging and Data Sciences, Faculty of Biology, Medicine and Health, Manchester Academic Health Science Centre, School of Health Sciences, University of Manchester, Manchester, United Kingdom; MRC Centre for Neuropsychiatric Genetics and Genomics, Division of Psychological Medicine and Clinical Neurosciences, Cardiff University, Cardiff, United Kingdom; School of Health and Wellbeing, University of Glasgow, Glasgow, United Kingdom; Department of Psychology, Institute of Psychiatry, Psychology & Neuroscience, King’s College London, London, United Kingdom; South London & Maudsley NHS Foundation Trust, London Hospital, London, United Kingdom; School of Health in Social Science, University of Edinburgh, Edinburgh, United Kingdom; School of Psychology, University of Sussex, Falmer, United Kingdom; Division of Psychology and Mental Health, Faculty of Biology, Medicine and Health, Manchester Academic Health Sciences, The University of Manchester, School of Health Sciences, Manchester, United Kingdom; Greater Manchester Mental Health NHS Foundation Trust, Manchester, United Kingdom; Division of Psychology and Mental Health, Faculty of Biology, Medicine and Health, Manchester Academic Health Sciences, The University of Manchester, School of Health Sciences, Manchester, United Kingdom; Greater Manchester Mental Health NHS Foundation Trust, Manchester, United Kingdom; School of Psychology, University of Sussex, Falmer, United Kingdom; Research and Development Department, Sussex Partnership NHS Foundation Trust, Hove, United Kingdom; School of Health and Wellbeing, University of Glasgow, Glasgow, United Kingdom; NHS Greater Glasgow & Clyde, Glasgow, United Kingdom; Division of Psychology and Mental Health, Faculty of Biology, Medicine and Health, Manchester Academic Health Sciences, The University of Manchester, School of Health Sciences, Manchester, United Kingdom; Greater Manchester Mental Health NHS Foundation Trust, Manchester, United Kingdom; MRC Centre for Neuropsychiatric Genetics and Genomics, Division of Psychological Medicine and Clinical Neurosciences, Cardiff University, Cardiff, United Kingdom; The McPin Foundation, London, United Kingdom; Division of Psychology and Mental Health, Faculty of Biology, Medicine and Health, Manchester Academic Health Sciences, The University of Manchester, School of Health Sciences, Manchester, United Kingdom; Division of Psychology and Mental Health, Faculty of Biology, Medicine and Health, Manchester Academic Health Sciences, The University of Manchester, School of Health Sciences, Manchester, United Kingdom; Greater Manchester Mental Health NHS Foundation Trust, Manchester, United Kingdom; School of Health in Social Science, University of Edinburgh, Edinburgh, United Kingdom; Centre for Youth Mental Health, University of Melbourne, Melbourne, Australia; Orygen, Melbourne, Australia; School of Health and Wellbeing, University of Glasgow, Glasgow, United Kingdom; Division of Psychology and Mental Health, Faculty of Biology, Medicine and Health, Manchester Academic Health Sciences, The University of Manchester, School of Health Sciences, Manchester, United Kingdom; MRC Centre for Neuropsychiatric Genetics and Genomics, Division of Psychological Medicine and Clinical Neurosciences, Cardiff University, Cardiff, United Kingdom; Division of Psychology and Mental Health, Faculty of Biology, Medicine and Health, Manchester Academic Health Sciences, The University of Manchester, School of Health Sciences, Manchester, United Kingdom; School of Health and Wellbeing, University of Glasgow, Glasgow, United Kingdom; School of Health in Social Science, University of Edinburgh, Edinburgh, United Kingdom; NHS Lothian, Edinburgh, United Kingdom; Research and Development Department, Sussex Partnership NHS Foundation Trust, Hove, United Kingdom; Division of Psychology and Mental Health, Faculty of Biology, Medicine and Health, Manchester Academic Health Sciences, The University of Manchester, School of Health Sciences, Manchester, United Kingdom; Research and Development Department, Sussex Partnership NHS Foundation Trust, Hove, United Kingdom; MRC Centre for Neuropsychiatric Genetics and Genomics, Division of Psychological Medicine and Clinical Neurosciences, Cardiff University, Cardiff, United Kingdom; Research and Development Department, Sussex Partnership NHS Foundation Trust, Hove, United Kingdom; Centre for Primary Care and Health Services Research, Division of Population Health, The University of Manchester, Manchester, United Kingdom; Department of Psychology, Institute of Psychiatry, Psychology & Neuroscience, King’s College London, London, United Kingdom; South London & Maudsley NHS Foundation Trust, London Hospital, London, United Kingdom; Department of Psychology, Institute of Psychiatry, Psychology & Neuroscience, King’s College London, London, United Kingdom; Division of Psychology and Mental Health, Faculty of Biology, Medicine and Health, Manchester Academic Health Sciences, The University of Manchester, School of Health Sciences, Manchester, United Kingdom; Division of Psychology and Mental Health, Faculty of Biology, Medicine and Health, Manchester Academic Health Sciences, The University of Manchester, School of Health Sciences, Manchester, United Kingdom; Greater Manchester Mental Health NHS Foundation Trust, Manchester, United Kingdom

**Keywords:** relapse prediction, active symptom monitoring, passive sensing, machine learning, staff views

## Abstract

**Background and Hypothesis:**

Digital remote monitoring (DRM) captures service users’ health-related data remotely using devices such as smartphones and wearables. Data can be analyzed using advanced statistical methods (eg, machine learning) and shared with clinicians to aid assessment of people with psychosis’ mental health, enabling timely intervention. Such methods show promise in detecting early signs of psychosis relapse. However, little is known about clinicians’ views on the use of DRM for psychosis. This study explores multi-disciplinary staff perspectives on using DRM in practice.

**Study Design:**

Fifty-nine mental health professionals were interviewed about their views on DRM in psychosis care. Interviews were analyzed using reflexive thematic analysis. *Study Results:* Five overarching themes were developed, each with subthemes: (1) the perceived value of digital remote monitoring; (2) clinicians’ trust in digital remote monitoring (3 subthemes); (3) service user factors (2 subthemes); (4) the technology-service user-clinician interface (2 subthemes); and (5) organizational context (2 subthemes).

**Conclusions:**

Participants saw the value of using DRM to detect early signs of relapse and to encourage service user self-reflection on symptoms. However, the accuracy of data collected, the impact of remote monitoring on therapeutic relationships, data privacy, and workload, responsibility and resource implications were key concerns. Policies and guidelines outlining clinicians’ roles in relation to DRM and comprehensive training on its use are essential to support its implementation in practice. Further evaluation regarding the impact of digital remote monitoring on service user outcomes, therapeutic relationships, clinical workflows, and service costs is needed.

## Introduction

Psychosis poses considerable personal and public health challenges, with schizophrenia being one of the leading causes of disability worldwide.^1^ Globally 71% of people with psychosis do not receive mental health care^2^ and relapse rates are high, with 36% of people experiencing a relapse within a year of their first episode.^3^ Relapses are associated with significant costs for mental health services.^4–7^ Current mental healthcare provision for people with psychosis is inadequate, with significant staff shortages across all mental healthcare professions resulting in long waiting lists and treatment delays that lead to crisis-driven and reactive care.^8,9^ In this landscape, it is challenging for mental health professionals to identify, and respond proactively to, early signs of people’s mental health deteriorating.

Digital technology is increasingly recognized as playing an important role in improving access to and the quality of mental healthcare, with organizational bodies, such as the World Health Organization, the UK National Institute for Health and Care Excellence, and the UK National Health Service (NHS) developing initiatives to support digital health development and deployment.^10–12^ One such technology, digital remote monitoring (DRM), has the potential to improve mental health by detecting early signs of relapse in people with psychosis.^13–15^ DRM involves regular symptom monitoring via digital devices and can include both active monitoring of symptoms, where people record their “real-time” symptoms through digital devices (eg, via questionnaires on a smartphone app), and passive collection of contextual health-related data, such as information on sleep, physical activity, and location, via sensors in a smartphone or wearable device.^15^ Collated data can be analyzed using machine-learning methods, which involve algorithms that detect patterns and make predictions from highly characterized datasets. This can then be shared with mental health services to support clinical assessment and identify priority areas for targeted intervention.^14^

DRM can provide rich information to supplement service user reports by collating near real-time data on changes in symptoms, mitigating recall biases evident in traditional clinical assessment methods.^16–18^ This is of particular value in psychosis, where cognitive difficulties commonly affect symptom recall and timely access to personalized interventions in response to early warning signs (EWS) is central to relapse prevention. Preliminary evidence suggests DRM can identify unique digital indicators for psychosis relapse^19,20^ and is feasible, acceptable, and safe for people with psychosis.^14^ Such findings suggest DRM could be used in clinical practice to identify EWS of relapse, enabling intervention before a full relapse occurs: this has the potential to significantly reduce the devastating personal, social, and economic costs of relapse.

Despite its potential, DRM approaches for psychosis are still evolving, and implementing novel digital technologies in real-world clinical settings is not without challenges.^21^ These include concerns about data privacy and security, trust from both clinicians and service users, varying levels of digital literacy, and maintaining sustained engagement with digital interventions, to name a few. As well as the importance of people with psychosis finding DRM usable, acceptable and effective, clinicians’ attitudes toward, and knowledge about, novel technologies have been found to influence their implementation in practice.^16,22^ It is essential to consult both service users and clinicians who are expected to use digital technologies in their day-to-day practice to ensure such technologies are workable and effective.^23^ Despite the growing interest in DRM within mental healthcare, several limitations and knowledge gaps exist regarding mental health professionals’ perspectives on its implementation in psychosis care. First, several existing studies use quantitative (survey) methods to explore digital mental health more broadly,^24–26^ which are not designed to capture the depth and nuance of clinicians’ experiences with DRM. Second, existing qualitative studies have primarily examined active symptom monitoring (ASM) and/or comprised small, localized samples,^27–30^ limiting the transferability of findings across geographical locations and diverse settings. Third, few studies have specifically examined passive sensing and machine learning approaches in the context of psychosis care.^31^ As a result, research to date has yet to fully explore the unique challenges and facilitators of DRM across diverse clinical settings. By addressing these gaps with a detailed, context-rich qualitative approach, we can gain a deeper and more nuanced understanding of clinicians’ perspectives. Therefore, this study aimed to qualitatively explore the perspectives of multi-disciplinary staff on the feasibility, benefits, and challenges of implementing DRM in mental health services for people with psychosis. Our focus is on identifying key factors influencing adoption, potential barriers, and facilitators, and how DRM could be integrated into existing care pathways across diverse geographical and service settings.

## Methods

### Study Design

This qualitative study was nested within a broader programme of work funded by The Wellcome Trust (www.connectdigitalstudy.com). The CONNECT cohort study aims to develop a digital platform used for predicting psychosis relapse, and an adaptive sampling framework that dynamically adapts the frequency of active symptom monitoring. To do this, data will be captured actively via a self-monitoring app, passively via sensors in a smartphones and/or a wearable devices, and from clinical assessments and medical records. Statistical and machine-learning methods (which use algorithms to identify patterns in data and make predictions based on past observations) will be used to analyze the data with the aim of creating a digital system that monitors service users’ mental health and alerts them and/or their clinical team to early signs of relapse, enabling timely access to support.

This qualitative study was underpinned by a critical realist epistemological position, which is particularly well-suited for exploring complex social phenomena such as the implementation of DRM in mental health services. Critical realism posits that there is both an objective social world and that one’s understanding of the social world is limited by one’s experiences and position within it.^32^ This position is particularly valuable in this study, as it enables an in-depth exploration of mental health professionals’ experiences, recognizing both their subjective interpretations and the structural and contextual factors influencing DRM adoption. Critical realism supports the identification of underlying factors that shape practice, allowing for nuanced insights that extend beyond individual accounts, allowing for tentative transferability of findings to similar settings.^32^ The study is reported in line with the Consolidated Criteria for Reporting Qualitative Studies checklist.^33^ Institutional and Health Research Authority ethical approvals were granted (REC Number: 22/WS/0083).

### Participants and Sampling

**Figure 1.**
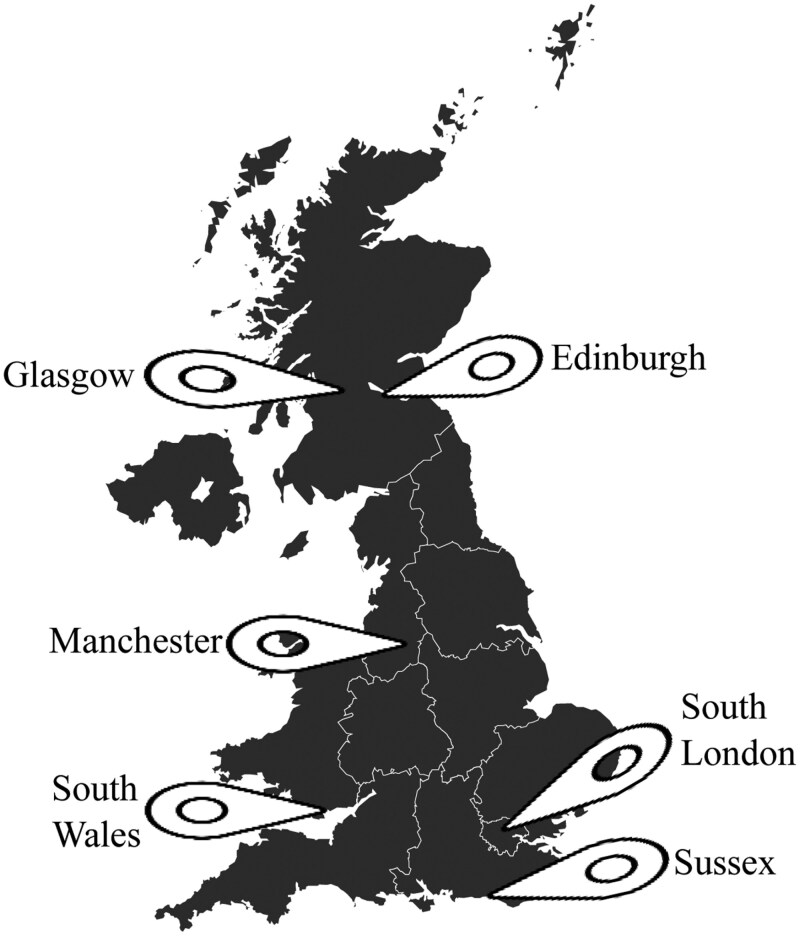
CONNECT study research sites.

Purposive sampling^34^ was used to recruit participants from 9 NHS mental health Trusts/Health Boards across 6 geographical sites in the United Kingdom: Manchester, Sussex, South London, Glasgow, Edinburgh, and South Wales (see Figure 1). Inclusion criteria included (1) staff working within an adult mental health service supporting people who experience psychosis; (2) a good understanding of the English language; and (3) ability to provide informed consent. Efforts were made to recruit staff with varying professional backgrounds to reflect the multi-disciplinary NHS workforce, including those in clinical roles as well as in leadership positions. This involved targeted invitations, engagement with different NHS teams, and recruitment across diverse clinical and managerial settings to ensure broad representation.

### Procedure and Data Collection

Researchers approached managers of services for permission to promote the study via posters, leaflets, emails, and attendance at team meetings. Interested staff contacted researchers for further information. Informed consent was obtained via signed paper consent form, signed electronic consent form, or audio recorded consent. Participants were assigned a unique ID number to ensure anonymity. Interviews were conducted one-to-one either in-person at the participant’s place of work or via videoconferencing software. Interviews were recorded using encrypted software and had an average duration of approximately 1 hour (ranging from 24 to 75 minutes). Each interview was transcribed verbatim, anonymized to protect participant confidentiality, and securely stored. All participants also completed a demographics form. Field notes and reflective logs were kept throughout data collection.

Interviews were based on a topic guide developed in consultation with clinical experts that was pilot tested to assess the language and clarity of the questions, and the flow of the interview. Questions addressed participants’ views on the use of DRM in the care of people with psychosis. Example questions included: *What would help you as a clinician feel more comfortable about health-related information being gathered in this way? What do you think about using these methods to spot early signs that a service user might be experiencing an impending psychosis relapse? What support would you/staff need to use a digital remote monitoring system like CONNECT?* DRM was defined as the use of both active symptom monitoring via an app and passive monitoring of health-related behavior via sensors in smartphones and wearable devices, as well as the use of a personalized algorithm developed using machine learning methods to detect, and alert clinicians to, EWS of psychosis relapse. These concepts were explained to participants using example scenarios during the interviews (see Text, Supplementary Data Content 1 for the topic guide). Questions were open-ended and probes were used to facilitate elaboration where applicable. The reflective logs were used to iterate the topic guide during the data collection period. For example, questions about the implications of DRM implementation at managerial level were added. Interview data were collected between November 2022 and January 2024.

### Data Analysis

Analysis was inductive, guided by reflexive thematic analysis protocol,^35^ and supported by NVivo qualitative data analysis software.^36^ Data analysis followed a 6-stage iterative process: (1) familiarization with the data, (2) code generation, (3) initial theme creation, (4) revision of themes, (5) defining final themes, and (6) writing the report.^35^ Two researchers (HB and XZ) coded the data; codes were discussed and compared as analysis progressed. Themes were primarily created by HB: they were reviewed and refined by EE, JN, PW, and SB to ensure they were reflective of the original data, related to the aim of the research and told a core interpretative story.^35,37^ There were no attempts to determine inter-rater reliability, in line with a non-positivist approach which embraces, rather than attempts to mitigate, researcher subjectivity^35,37^ (see Text, Supplementary Data Content 2 for further information on reflexivity).

## Results

### Participant Characteristics

Ninety-one staff expressed an interest in taking part: most of those who subsequently did not participate failed to respond to initial enquiry, others declined due to time constraints or role changes. The final sample (*n* = 59) included 12 participants in leadership positions and 47 employed in traditionally more service-user facing roles (eg, psychologists, psychiatrists, mental health nurses, social workers, support workers). See Table 1 for further participant characteristics.

**Table 1.** Participant Characteristics.

Gender	*N* (%)
Female	38 (67)
Male	21 (33)
**Ethnicity**	
White British	46 (78)
White Other	8 (13)
Black African	2 (3)
Chinese	1 (2)
Indian	1 (2)
Other Asian Background	1 (2)
**Mean age in years (range)**	41.48 (25–66)
**Service area**	
Community Mental Health Team	22 (37)
Early Intervention in Psychosis Team	15 (26)
Inpatient Services	10 (17)
Mental Health Liaison Team	4 (7)
Assertive Outreach Team	3 (5)
Specialist services	3 (5)
Cross-service leadership	2 (3)
**Mean (range) years working for service**	5.3 (0.1–26)
**Mean (range) years working in mental health services**	15.2 (0.3–42)
**Discipline**	
Psychiatrist	15 (25)
Psychologist	10 (17)
Team Manager / Service Lead	12 (20)
Mental Health Nurse	7 (12)
Social Worker	7 (12)
Support Worker	2 (3)
Employment Specialist	1 (2)
Junior Doctor	1 (2)
Mental Health and Wellbeing Practitioner	1 (2)
Peer Support Worker	1 (2)
Occupational Therapist	1 (2)
Nurse Advanced Practitioner	1 (2)

### Themes and Subthemes

Five overarching themes and 9 corresponding subthemes (Table 2) were developed. Supporting quotes are presented in Table 3.

**Table 2.** Themes and Subthemes.

Themes	Subthemes
1.The perceived value of digital remote monitoring	
2. Clinicians’ trust in digital remote monitoring	2.1 Clinician digital confidence and competence
	2.2 Privacy issues
	2.3 The quality and accuracy of the data
3. Service user factors	3.1 The “right type” of service user
	3.2 Issues of (ongoing) consent
4. The technology-service user-clinician interface	4.1 The relational context
	4.2 Risks and responsibilities
5. Organizational context	5.1 Stretched services and stretched clinicians
	5.2 Embedding DRM into service delivery

**Table 3.** Themes, Subthemes, and Supporting Quotes.

Theme	Subtheme	Supporting quotes
1.The perceived value of DRM		*“We might miss these prodromal symptoms or some relapse signs, just because we’re not in the position to see the service user often enough or maybe when they are unwell that’s when they don’t want to get in contact with services. So, I think that if we have an alternative to get this information, it would be very, very useful.” (K501)* *“I suppose if perhaps the person that you are working with is not… being open or is not telling you what’s going on if they are having some difficulties…then this would almost be an objective way of showing that there are some issues and communicating that to you.” (M509)* *“The benefits would be…building evidence for [service users] and helping their insight…I think if they can…self-report ‘I was feeling panicky at this time in the day’ and then the data shows that you were on a phone call, we can say ‘well what were you doing during that, that phone call?’, ‘I was having a really difficult conversation with my boss’, then they could kind of make sense as to why they were feeling panicky without needing to think back retrospectively to what, what was going on.” (S506)* *“It would be quite good for people with…bipolar disorder or mood difficulties, where there will be changes in sleep, activity, these sorts of things are definitely, maybe, pre-cursors to becoming more unwell. But I think for the psychosis group it can be…a florid relapse quite quickly…So I’m not sure how helpful [DRM would be].” (G502)* *“…it’s one of the biggest factors of service users’ health, is their physical health. I think some of that with the smart wearable stuff…[could] be really useful to monitor their physical health. But yes, it would worry me about monitoring a smartphone…it feels a bit too much, really.” (C503)*
2.Clinicians’ trust in digital remote monitoring	2.1 Clinician digital confidence and competence	*“We don’t get guidance as to specific apps for individuals to access...there’s [not] a gold standard, or a…recommended app.” (C501*) *“We’d have to be shown how to use it, to understand it, how to decipher the data. If it went wrong, what would we do? We’d probably need an understanding of who we can contact if something went wrong with it...I struggle with technology, always have done… But with the support, I’d definitely give it a try.” (M508)* *“I notice often when new technology is implemented…people are expected to implement new systems…on the top of their existing workload and also by themselves. So, it’s a lot of self-directed learning…I think sometimes it’s over-estimated how much time and energy and skills [clinicians] already have to implement some things...So, it would be great to think [about] how clinicians can be eased into using these and being trained and being supported and having access to additional support throughout.” (E501)*
	2.2Privacy issues	*“It’s always about privacy and people’s right to do whatever…I think particularly for patients who are detained…or who are on a [community treatment] order I think they would be very suspicious about use of technology in that way, as another form of control.” (G508)* *“I don’t really think [DRM data] should be sent to the teams though…I personally would be a bit like that’s a bit too much invasion of privacy…That is too much for me. But again, it’s an individual choice.” (M503)* *“If [service users] ask us questions [such as] ‘Where is my information going? Why is this information being taken?’. If we can’t answer that, then I wouldn’t feel comfortable suggesting it.” (S501)* *“Data protection would be…a very important issue for me. [I would need] some convincing information about the storage of personal information about the patient…it would need to be convincing and so it would need to be explained to the patient very easily for him to consent.” (K503)*
	2.3 The quality and accuracy of the data	*“[Passive data] would really need to be put into some form of context...it could be something completely innocent, that…for example, if the device indicates they haven’t been outside the front door for X amount of days, or their step rate has gone down dramatically, there could be a multitude of completely innocent reasons why…any information really has to be contextualized.” (C501)* *“There is a risk of some people being overtreated, basically, because you could say, ‘oh, this sounds like you might be relapsing, things might be going downhill a bit, maybe we will increase the dose of your antipsychotics’, when that might not have happened, and they might have been fine.” (M504)* *“I think people will still be meeting people, they’ll still go with their gut, they’ll still use their skills that a computer will never have. [DRM] will be helpful, but it won’t be the ‘be all and end all’. I think that we will continue to work around it, and it will be helpful when it works and, when it doesn’t, we’ll continue working in the way that we do and that’s OK.” (E507)* *“It depends on the quality of the [relapse prediction algorithm] tool because if you’ve just got a spectacular number of false positives…if it’s wrongly saying, you know, ‘everyone is relapsing’ then…you’d get a bit fed up of the tool, I suspect. And then you’d probably just disregard it…if it was wrong all the time.” (K505)*
3.Service user factors	3.1 The “right type” of service user	*“…there’s a big chunk of the client group that I work with that really don’t like technology…and it’s really tricky, even using their phones, they really struggle with that. But there are some folk that have worked with a few people that have used [wearable devices] and they’re familiar with phones…so yeah, I guess there would be few folk that would probably take to [DRM] quite easily.” (E509)* *“…the younger generation have grown up with digital and mobiles and so they might be more competent with using a mobile phone and using apps…they’re a lot more transparent and open and engaged with [data sharing] …they think everybody knows everything about them anyway so what does it really matter? And they’re, kind of, open to that mentality.” (M512)* *“[Using technology to] monitor and track how [service users are] doing could actually potentially trigger a relapse if…if it becomes entwined in any sort of delusional belief system they have, or if they start to become paranoid or suspicious of it, and that could actually be harmful for the therapeutic relationship with the mental health service as well.” (K510)* *“It’s within their presentation, they’ve got a particular bias, as it were…I think the more manipulative might want to use [DRM] as a way of benefitting themselves, as it were, getting themselves a quick admission to hospital to avoid their drug debts, ‘oh, look I’m relapsing and this, this, and this, so I need to be in hospital,’ you know?” (M506)*
	3.2 Issues of (ongoing) consent	*“I think if they’ve made an informed decision about it, and they want [DRM] to be part of their care package, that’s fine…there’s probably a percentage of our folk that won’t fully understand the technology and might not be able to make a properly informed decision.” (E504)* *“I think my gut reaction is that [DRM] could be so helpful but the patients that it would be useful and helpful for wouldn’t probably be the ones that could give informed consent for it…I think there would need to be very stringent safeguards around its use in insight-limiting illnesses.” (G505)* *“…I don’t think you could section someone and force them to wear a wearable device. That’s just wrong. You can’t make people do stuff, everyone has bodily autonomy. That’s a moral right that’s inherent to being a human being.” (K504)*
4.The technology-service user-clinician interface	4.1 The relational context	*“I always am a little bit worried about using technology because it can actually lead to people feeling disconnected, because maybe they wouldn’t speak to their care coordinator…if they’re reporting things on an app more…and that could lead to like a disconnection with the service.” (K510)* *“…if you have this app system and you know you [visit people] weekly at first, but actually [the app is saying] ‘they’re fine and they don’t really need intensive support’…we’ll take our foot off the pedal and focus more on other people…and then in two months’ time and it’s flashing at you saying ‘everything’s awful’…things were going awry for weeks beforehand, but because you were relying on the app to alert you to anything wrong [you didn’t know]…you lose that kind of therapeutic alliance and relationship building.” (G507)* *“This could be a really great way of starting a conversation, ‘look, that app that you agreed to is telling us that you are struggling to sleep recently’…almost like a good starting point for a conversation…around maybe some factors that we weren’t aware of. The person’s not telling us that they’re not sleeping…so maybe this can help with that kind of thing, like being aware of more factors that potentially are affecting their health.” (M511)*
	4.2 Risks and responsibilities	*“[Clinicians] need to respond to [DRM data] because if they don’t and somebody…you know, an incident occurs, then they’ve not responded to the information that they have sight of… the most extreme [consideration] is fear…is clinical safety, is accountability, responsibility…lack of response and ending up in coroner’s [court] as a consequence of not responding to the right information at the right time.” (M512)* *“We would all need to be clear, as to what we then would do, as a service…if that information [from DRM] comes to us…with care plans we provide now, paper care plans, we have a crisis contingency plan…everybody knows in advance, if X happens, or Y happens, there will be Z response…that would need to be the same again [when using DRM], wouldn’t it?” (C501)* *“My only reservation would be, it’s almost handing over responsibility for, you know, seeking help and contacting services then to a third party. And that can be something that we really… it can take a lot of work to try and build up a sense of taking responsibility for your own mental health that I think is really important for longer term recovery.” (G505)*
5.Organizational context	5.1 Stretched services and stretched clinicians	*“There could be a bit of resistance, that people would see [DRM] as yet another thing that they’re having to do in their daily work, or understand, or work with, that might feel like an additional pressure...when services are already really pressured.” (G510)* *“I suppose it’s just that the idea that [DRM] is an extra thing that could be complicated, that could take up a lot of time, [we’re] going to have to look through that data, make sense of that…if it adds to workload…getting to grips with anything new initially can take a wee bit of time to incorporate into your work so that’s really the main barrier I think probably, or perceived barrier.” (E509)* *“The NHS computer systems and general IT setup is pretty terrible…I think actually having access to hardware and software that functions reliably so that you can access [DRM] and use it…that’s a real challenge.” (S509)* *“I guess one thing that comes to mind first is the cost…budgets are very, very tight…how realistic is it to be able to implement [DRM] in terms of funding and budget? I think that’s, the practical hurdle…realistically that’s something that would come up.” (K510)*
	5.2 Embedding DRM into service delivery	*“Even finding somebody…an actual team member who might be really interested in [DRM]…be the kind of go-to person on the team level…we’ve used that kind of model for other things and that’s been helpful.” (G510)* *“If we got an alert that somebody may be relapsing it would go to the duty worker and the duty worker would give them a call and see how they are…” (E507)* *“If the data was easier to record, accessible, you could get a nice graph from it and it could…go in their electronic notes…all the services could see it, [that would be] much more useful [than paper notes].” (E509)* *“The biggest thing is making sure…the creation of the app and how it’s delivered through whichever system…making sure [clinicians are] involved in the process…having it being developed by them for them… if it’s designed for you by you it’s a bit different than someone saying, “oh here use this.”...and you’re like, “but this doesn’t help me in my day-to-day [work] this is what I actually want to see, not this.” (K507)* *“You’d have to show…the benefits of [DRM], so people are interested if they think it will help their patients…if people thought it was just a number crunching exercise or something for the Trust or the NHS they wouldn’t [be] interested.” (S510)*

### Theme 1. The Perceived Value of Digital Remote Monitoring

Most participants perceived the main benefit of DRM was that it could provide another source of information for assessment and care planning, deemed particularly useful for identifying patterns in service users’ symptoms and recognizing EWS for relapse. Participants thought having this additional information could facilitate prioritzsation of care and help clinical teams to *“be more responsive and get in quicker…[which] might avoid a hospital admission” (E509).* Some participants struggled to see how DRM would benefit people with “typical” nonaffective psychosis, instead seeing more value in using DRM for mood difficulties. There was a sense this was because psychosis relapse was perceived to be quick and unpredictable, and thus would be ineffectively captured by remote monitoring:

*“It would be quite good for people with…bipolar disorder or mood difficulties, where there will be changes in sleep, activity, these sorts of things are definitely, maybe, pre-cursors to becoming more unwell. But I think for the psychosis group it can be…a florid relapse quite quickly…So I’m not sure how helpful [DRM would be].” (G502)*

DRM data, specifically passively collected data, was perceived to be more objective than traditional assessment methods that rely on service user reports. Participants also noted continuous passive data collection provides information about service users’ lives at times when clinicians are not present, which was deemed particularly helpful for service users who have limited contact with services. Several participants felt that passive data collection would therefore provide evidence about service users’ activity and symptoms, where “*if people say, ‘I haven’t left the house for a week’…you wouldn’t just have to take their word for it, you might have some evidence of that” (S505*). Participants thought such factors could enhance the validity of assessments, enabling more effective clinical decision-making.

Many participants also thought DRM, particularly active symptom monitoring, could encourage service users to reflect on their experiences. It was noted that service users can have difficulty recalling their experiences during appointments due to memory difficulties or difficulties engaging with the clinical team. The information collected by DRM, alongside support from clinicians, could help service users to develop “insight” into their mental health that could ultimately help them better manage their experiences:

*“…the more chaotic patients, if they’re manic, if they’re hearing voices a lot, they struggle to be able to reflect on what they’ve got up to in a day, especially in a week…if this information could be presented back, ‘actually, you’ve only slept for three hours per day all week’…it’s a good way of being able to say, ‘this is how you’ve been’.” (M508)*

In general, participants welcomed the use of active symptom monitoring to provide information about service users’ mental health. However, passive data collection was seen to be most useful and valid for physical health monitoring, with less understanding of how it may provide insights into mental health. The perceived legitimacy of physical health monitoring for this client group meant many clinicians appeared more comfortable with collecting passive data for physical health rather than mental health purposes:

*“But I think in terms of the physical health side of things it’s a lot easier than the mental health side of things… I think ‘cause we’re looking at tangible results, as it were. So, you can see the number of steps, or you can chart the number of calories that have been burnt off, you can look at the extent of the activity” (M506).*

### Theme 2. Clinicians’ Trust in Digital Remote Monitoring

#### Clinician Digital Confidence and Competence

Participants thought that some clinicians lack confidence and experience with apps and wearables in their clinical practice due to unfamiliarity, limited device access, and low awareness of NHS-approved digital innovations. Some participants spoke about how a lack of confidence in technology would likely impact on clinicians’ ability or inclination to use DRM in practice, where “*trying to bring in an app where they have to potentially check in and log in with what other people are doing and read that data might be really tricky.” (S502*).

Given such concerns, participants highlighted the need for clinicians to be reassured that DRM is evidence-based, clinically effective, “*that it’s tried and tested, that people have used it, it is working” (C503)* before they would consider using it. Participants reported that staff training to increase staff confidence, knowledge and trust in DRM would be crucial for adopting it in practice. Training topics should include how to use the digital devices, how to interpret the data, and data storage and sharing arrangements. Many participants also felt that clinicians would require ongoing support to effectively use DRM including having access to individuals with digital expertise who can assist with troubleshooting.

#### Privacy Issues

Many participants felt that the amount and types of data collected by DRM was excessive and considered sharing this data with clinicians to be an invasion of service users’ privacy, particularly sharing passively collected data. There was a sense this could potentially exacerbate power imbalances between clinicians and service users:

*“I wouldn’t want that information going to my mental health team …it almost feels like, you know, you’re an adult and someone is babysitting you.” (S504)*

Passively collected location data appeared to cause the most concern for clinicians, particularly if it involved the sharing of service users’ location with the clinical team. Although some clinicians highlighted the utility of knowing service users’ whereabouts in crisis situations, most participants did not see location data as clinically useful or justifiable to collect. Some participants described tensions between needing to work in a “least restrictive way” and the potential “*pressure on clinicians to use [location data] …if anything goes wrong it’s a question of ‘why didn’t you use it?’ So, it potentially would lead to quite an increase in some restricted and coercive practice.” (M504*)

In general, concerns about privacy appeared to be less of an issue for actively collected data, perhaps because this requires direct input from the service user, implying a more concrete decision to share this information. Whether collected actively or passively, almost all participants spoke about the importance of data being stored safely and securely, with some expressing concerns about the potential for data breaches. While some participants felt reassured that data would be stored appropriately due to legal and organizational governance requirements, a larger number expressed concerns this might not be the case. This uncertainty affected their trust in DRM, and some participants felt they did not understand DRM and data sharing enough to use it in their work. Participants emphasized the need for clear information about where data would be stored, who it would be shared with, and the reasons for sharing it and stated this information should be provided in an accessible format for service users to ensure they understand what will happen with their data.

#### The Quality and Accuracy of the Data

Clinicians’ trust in DRM was affected by the perceived quality and accuracy of the data. For example, some participants noted that feedback from their own wearables can inaccurately reflect their behaviors, causing them to question the accuracy of data collected in this way. Participants also questioned the comprehensiveness of the data because they felt service users would be unlikely to consistently engage with DRM due to amotivation and cognitive difficulties, meaning “*if someone is presenting with early warning signs, we might miss the actual timescale because they may not be filling in the questionnaire.” (K509*)

Most participants highlighted that having de-contextualized passive data about service users (e.g., about their sleep or activity) without knowing the reason for any change in these measures, limited the utility of the information. Many spoke about the “innocent” reasons as to why a change might be detected by the DRM system and the potential for staff to respond with unnecessary interventions if they were to rely on this data alone to inform their clinical decision making. Thus, they emphasized the importance of considering DRM data alongside their own clinical judgement, rather than replacing it:

*“…don’t just ignore your own training and your own experience and say, ‘OK, well, the machine has told us that, so that must be true’…you only need to look at a weather forecast sometimes, and it’ll say, ‘it’s bright and sunny today’ and there’s hail as you speak…so don’t replace your own judgement.” (K505)*

Since participants generally viewed DRM as a supplementary source of information, they broadly accepted relapse prediction algorithms might not always be accurate and acknowledged that clinicians could receive both false-positive and false-negative relapse alerts. However, there was a limit to this. Participants expressed concerns that false-positive relapse alerts (ie, where the algorithm identifies someone to be relapsing when they are not) could worry service users and inadvertently lead to a relapse that would not have happened otherwise. Others noted that if DRM is too inaccurate clinicians might dismiss the alert altogether and “*lose faith in it.” (E503)*

### Theme 3. Service User Factors

#### The “right type” of Service User

Participants expressed their views on which service users DRM is appropriate for. Many said that people with psychosis often experience difficulties in accessing and using digital devices due to poverty and lower levels of digital literacy, making it challenging for these service users to adopt DRM. Most participants stated that they felt DRM would be more appealing to younger service users because they were considered more likely to already regularly engage with technology and to be more accustomed to sharing data online. Overall, DRM was typically seen as more appropriate for service users who usually use digital devices and were motivated to do so.

Almost all participants expressed that DRM may not be appropriate for people experiencing paranoia: there was a sense participants would feel uncomfortable broaching the subject of using digital devices with such service users due to fears DRM might distress them. Additionally, some staff expressed concerns that DRM could exacerbate paranoia:

*“… [service users] can be quite worried about who’s watching them…And if we’re actually doing that to patients, then it enforces a lot of the beliefs that are already there…they might become more paranoid...I don’t think you’d even bother offering them [DRM], if they felt [paranoid].” (C503)*

There were also concerns that DRM, particularly the active monitoring of symptoms, may cause some service users to become “obsessive” about tracking their mood and health-related behavior, which could make them more anxious and increase rumination on negative experiences. As such, participants perceived DRM to be less appropriate for “*anybody with a significant risk history...where there are fluctuations in mood that happen very quick.” (S506)*

Furthermore, some participants believed that certain service users may be dishonest in their reporting of symptoms, either over-reporting to access further support or under-reporting to avoid interventions by clinicians. Others questioned whether some might deliberately skew passive data collection; for example, by having others wear wearables for them or by only wearing wearables at certain times. This fed back into concerns about the accuracy of DRM data and participants were hesitant to use DRM with service users they believed might manipulate the data in this way.

#### Issues of (ongoing) Consent

Participants stated it was necessary for service users to provide informed consent to use DRM, noting this would require a thorough understanding of DRM and how it works, with some participants thinking service users may not understand the technology enough to be able to consent to using it. Participants also spoke about consent needing to be revisited with service users because their capacity or choice to consent to DRM may fluctuate, particularly if they are relapsing. Some participants thought this would lead to ethical and legal ambiguity about whether DRM could still be used in such circumstances. They argued this would limit the usefulness DRM because people might not consent to using it during periods of more severe symptoms, precisely when the data would be most useful:

*“If a person is quite unwell…are they actually able to give consent right now? The consent would probably have to be given in advance of these technologies or permissions being put to use… Then what if they change their mind when they are unwell and say, ‘I don’t want you tracking where I am’. What does the team do then?” (G504)*

All participants felt strongly that service users should be able to opt in and out of using DRM. Although some participants acknowledged that other parts of healthcare can be enforced without service users’ consent under the Mental Health Act,^38^ all were clear that DRM should not be enforced under any circumstances. Reasons for this included concerns that mandatory use of DRM would unfairly exclude people from services; that many service users would be unable, or not want, to use the technology; that it would negatively affect service users’ trust and engagement with services; and that it would be restrictive and coercive.

### Theme 4. The Technology-service User-clinician Interface

#### The Relational Context

The impact of introducing DRM into therapeutic relationships was consistently discussed. Although most participants saw value in the information DRM could gather, participants believed that it cannot provide the “human factor” that therapeutic relationships provide:

*“[it is] not a substitute for a therapeutic relationship based on trust and judicial [sic] use of self as a clinician and mutuality that you get in a genuine therapeutic relationship.” (S508)*

Some participants thought that the consistent input from DRM could help service users feel cared for at times when not seeing clinicians, whereas others thought that there was a risk DRM could be used to replace therapeutic contact, leading to disconnection between staff and service users. Many participants felt that DRM data would be useful to bring into conversations with service users to facilitate care planning. This was deemed to be particularly useful where service users struggle to “open up” to the clinical team, where discussing data could be a “way in” for clinicians to talk to service users about their experiences. Conversely, some participants reported feeling uncomfortable about discussing information they knew about the service user that they had not explicitly shared themselves. This was particularly the case when DRM data may appear to contradict service user reports, with some participants worrying this could fuel mistrust between service users and clinicians:

*“…if you went in [to an appointment] saying, ‘do you know what, I already know you’re struggling’…‘we’re going to start tracking you…we’re going to challenge you on that’…they’d feel very, ‘well you don’t trust in what I’m telling you anyway’. It could break down your relationship.” (M508)*

Therefore, participants emphasized the importance of using DRM in a personalized manner within the context of an existing trusting, collaborative therapeutic relationship, “*where [DRM] really cements and grows the relationship, the trust, the decision making…” (M513)*

#### Risks and Responsibilities

Gathering remote data from service users was perceived to come with additional risks and responsibilities for clinicians. All staff noted that there would be a responsibility to respond to information received by DRM, particularly relapse alerts, and many expressed concerns about the consequences of not responding, such as professional liability and negative impacts on therapeutic relationships. One of the main concerns was that risk information relayed by service users via DRM might not be responded to in time to prevent serious incidents. Given these concerns, many participants queried where responsibility lies if they used data from DRM to inform their clinical decision making:

*“…If I base any of my actions on the results on this app and the results of the app are wrong, whose responsibility is it?…[if I] use this to inform my decision and I make a wrong decision…at least in part because of the output of the system, then whose responsibility is it?” (E506)*

Almost all participants emphasized the importance of clear organizational policies regarding responding to DRM data to help mitigate clinicians’ concerns and safeguard professionals. Participants also advocated for the use of response plans with service users to ensure they understand how their data will be used by services. Some participants talked about a potential shift in responsibility that DRM, specifically the use of relapse alerts, could cause, with worries that being notified about changes in people’s mental health takes the responsibility to manage this away from the service user. This was viewed as disempowering:

*“[clinicians] want service users to be less reliant on us…we want them to manage independently.” (C503)*

In contrast, service users actively tracking their symptoms was perceived to promote personal agency and help them feel less like passive recipients of clinical care. Thus, whilst some participants thought a digital system automatically alerting clinicians to potential relapse would be disempowering, it was thought that independent self-monitoring *“could be encouraging independence…[service users] taking control over their own recovery, and trying to identify their own symptoms, their own relapse indicators and managing and keeping on top of their own well-being…giving them a bit of autonomy and give them power over that.” (K510*)

### Theme 5. Organizational Context

#### Stretched Services and Stretched Clinicians

Throughout interviews, participants referenced the impact that the broader organizational system would have on the use of DRM in services. Participants consistently spoke about services being under-resourced, with most thinking that a DRM system would increase clinician workload due to the requirement to check the system and respond to alerts, noting “*if you’re under-staffed and overwhelmed, sometimes more information isn’t as helpful as it might sound.” (S501*)

Others thought that if clinicians found the initial effort and time required to adopt DRM manageable, then it could have benefits longer term by helping staff to manage their caseloads through enabling prioritization of care. The importance of data from DRM being quick and easy to interpret was highlighted, as it would allow clinicians to make timely clinical decisions:

*“…it’s just impossible for [clinicians] to keep track of everybody…if a CPN could spend half an hour looking at 20 patients on the screen and looking at a series of graphs… and go, ‘yeah, that’s fine, that’s usual for this person’, then [DRM] might well help with managing caseloads.” (E503)*

Many participants also commented on the current lack of financial resources and IT infrastructure in services that made it difficult to complete their routine work. In this context, it was difficult for participants to see how DRM could be implemented given the funding and IT infrastructure it would require.

#### Embedding DRM into Service Delivery

When considering what could help embed DRM into service delivery, many participants spoke about the senior leaders of organizations leading implementation from a “top down” approach, where it would become a normalized “*part of the culture of service delivery…very much a treatment option for patients” (G510).* Some felt that this would require key instigators in services, such as specialized “champions” or particularly motivated team members, to lead the way in promoting the use of DRM and to provide ongoing support to staff and service users.

Some participants suggested that DRM data should be integrated into multi-disciplinary team meetings, where it could provide supplementary information to aid team decision making. Several participants also thought that DRM, particularly relapse alerts, could be managed and responded to by duty workers, ie, clinicians assigned to respond to unscheduled contacts from service users and provide urgent care where necessary. On a practical level, participants spoke about factors that would make DRM easier to embed in services, such as ease of use, easily interpretable data, and automatic integration into clinical notes. Some participants mentioned that coproduction with both clinicians and service users would ensure such factors are considered in the development of DRM, making it more likely to be adopted by services.

Ensuring the benefits of DRM are known at organizational, clinician and service user level was considered to be one of the main facilitators to implementing DRM. Many participants noted that clinicians would be more likely to use DRM if it has positive clinical effects such as reduced relapse rates for service users. Ultimately, to get NHS organizations on board, DRM must save money by reducing crisis care and unnecessary clinical contacts:

*“…things are cost driven, so if there was a way that we can save on like staff time and resources…[if] it could potentially reduce hospital admissions…they cost money…they are hugely distressing for patients…if there was any impact on admissions that would be monumental for the NHS.” (C506)*

## Discussion

This is the first study of its size and scope to capture the nuanced perspectives of mental health professionals in both clinical and leadership roles regarding the use of DRM in psychosis care. This comprehensive account of diverse views will inform the development and implementation of DRM in routine practice.

Consistent with previous findings,^28,31^ participants in our study saw the value of DRM for identifying EWS of psychosis relapse and reducing the use of crisis interventions. DRM was perceived to provide a comprehensive clinical picture than traditional assessment methods through continuous collection of objective data that relies less on service user reports. Participants also expressed that DRM could increase service users’ understanding of their mental health and foster a sense of empowerment, a finding supported by first-hand accounts by people with psychosis.^39,40^ Despite its potential, participants expressed some concerns about DRM. In line with previous research on digital interventions for people with psychosis, privacy, and data sharing concerns,^41–43^ digital poverty and poorer digital literacy amongst service users,^28,36,38^ worries about the interplay between technology and paranoia and potential distress resulting from increased self-monitoring,^44,45^ the impact on clinician workload,^41,46^ concerns about risks and responsibilities^41,45^ and the potential for digital technology to neglect the therapeutic aspects of in-person healthcare^31^ were consistently discussed.

Our findings add to previous literature by highlighting issues to consider regarding the use of passive sensing and relapse prediction algorithms in psychosis care. Ongoing service user consent to passive data collection was a prominent issue for participants due to concerns that capacity to consent can fluctuate: participants expressed ethical concerns regarding continuing to use DRM in such circumstances. Initiatives such as dynamic consent^47^ or advanced directives^48^ could help provide clarity on consent processes for clinicians and empower service user decision making regarding the use of DRM in their care. Additionally, many participants believed that continuous monitoring of health-related data, particularly location data, is excessive and invades service users’ right to privacy. However, most proposals for using passive monitoring in mental healthcare do not involve the sharing of actual location data, rather location derived metrics (eg, location variance, entropy, and circadian movement) to ascertain changes in individual’s mental health^49^: clinician and service user views on the use of such metrics in the context of psychosis care should be further investigated.

Furthermore, participants were generally accepting of receiving passively collected physical health-related data (eg, on service user activity, sleep), reflecting findings from the general population also showing that people are more comfortable with sharing health-related data with healthcare professionals than personal data (eg, on location, social activity and communication).^50^ Service users’ sleep, activity and mood are commonly discussed in service user-clinician relationships: passive data reflecting this information is therefore more closely aligned to the norms of participants’ clinical practice,^50^ perhaps resulting in clinicians feeling this type of data is less invasive of service users’ privacy. There was also a sense that participants perceived health-related data to be more directly related to physical health than mental health and therefore more justifiable to collect. This could be reflective of wider westernized healthcare which encourages mind and body to be viewed as separate entities,^51^ potentially resulting in professionals devaluing the role behavioral or physical measures can play in informing mental health outcomes. Clinicians will need to be aware of how each type of data collected via DRM contributes to the assessment of service users’ mental health to understand its utility. Future research should consider how differences in the type of passive data both shared and received may influence service user and clinician engagement with DRM in practice.

Participants in our study appeared to understand the theoretical value of using a relapse prediction algorithm to identify, and alert clinicians to, EWS of relapse. However, some participants felt this would not effectively detect EWS due to beliefs that deterioration happens rapidly. There is some evidence that clinicians can confuse EWS of relapse with the initial symptoms of psychosis rather than recognizing them as affective, cognitive or basic symptom precursors.^52^ Some clinicians may therefore fail to appreciate the existence of EWS and thus undervalue the use of DRM for psychosis. Additional training in relapse assessment may therefore be important in supporting the adoption of DRM in services.

Furthermore, although previous research has considered the impact of digital monitoring on clinicians’ roles, responsibilities and workload,^41,46,53^ our findings highlight unique factors to consider in the context of receiving alerts from a machine learnt relapse prediction algorithm. Concerns about the accuracy of the data fed into worries that a relapse alert system would result in unsustainable increases in workload due to false positive relapse alerts. Although DRM has been posited to reduce clinician workload by aiding targeted intervention, the initial effort to understand it, adopt it and evaluate its effectiveness, and both the actual and conceptual shift from reactive to proactive care it would require, are likely barriers to its implementation.

Participants also expressed concerns about their responsibilities and professional liability if relapse alerts were inaccurate or missed. Such concerns echo previous findings that worries about risks can be a barrier to implementing digital interventions for people with psychosis^41,45^ and more broadly.^16^ Given these concerns, some participants expressed a preference for choosing when to access DRM data themselves rather than being automatically notified about potential EWS of relapse, perhaps because having this awareness comes with responsibility to act on it, which was perceived as not always possible in the context of an under-resourced system. Adopting an algorithm-based relapse alert system in services will require clear guidelines regarding the integration of DRM data into clinical decision making and clinicians’ professional responsibilities in relation to responding to alerts.

Despite the accuracy of relapse alerts being a concern, participants generally accepted some degree of inaccuracy, based on the understanding that clinicians would not rely solely on this data for clinical decisions. Imperfect data derived from DRM can still be valuable in guiding clinicians to consider certain possibilities and should be viewed as a tool to augment, not replace, clinician practice.^54^ This is important where the perceived objectivity and validity of DRM data could risk the exacerbation of epistemic injustice,^55^ where service users’ accounts of their experiences are dismissed due to unjustified preconceptions that they are not able to provide reliable knowledge.^56^ Clinicians should avoid over-determining what DRM data may mean in relation to a service users’ mental health^57^ and work collaboratively with service users when using DRM to inform clinical practice. Indeed, the sharing of data from DRM could facilitate communication between service users and clinicians: when used collaboratively, digital interventions have been found to enhance service user–clinician relationships.^58^

### Strengths and Limitations

A key strength of this study is our systematic approach, along with a large sample that captures perspectives from a broad geographical area. Our findings identify specific barriers, facilitators and contextual factors influencing adoption, providing actionable insights for policy and practice. This supports future digital interventions to better align with the needs and concerns of mental health professionals, who play a critical role in their implementation. There are, however, some limitations. Study participants were self-selected and therefore may hold stronger views about the use of technology in mental healthcare that may not be representative of the broader workforce. Despite aiming to recruit a diverse sample, the low representation of participants from ethnic minority backgrounds reflects the demographic composition of the workforce within the services we recruited from. Although there were no observable differences in perspectives across different geographical sites, professions, or services, we did not explicitly explore the ways in which these factors may influence participants’ viewpoints. Future research should consider whether these factors, as well as demographics such as age and ethnicity influence professionals’ perspectives. Additionally, given that a considerable barrier to implementing digital innovations in mental healthcare is user perspectives,^16^ views of other stakeholders, such as people with psychosis and policy makers, should be sought.

## Conclusions

Findings suggest that mental health professionals see the value in using DRM to support the care of people with psychosis, particularly to encourage self-reflection and to detect EWS. Nevertheless, they have realistic concerns about factors such as DRM’s evidence base and limitations, the service user–clinician relationship, service user consent, clinicians’ responsibilities and workloads, and organizational culture and resources. Training and supporting clinicians in using DRM is critical. Comprehensive guidelines and policies are needed to clarify the responsibilities of service users, clinicians, and organizations regarding the use of DRM in psychosis care. These guidelines should address risk management, service user rights, and data security and sharing procedures. Wider implementation of DRM will allow for evaluation of the cost-effectiveness of DRM as well as its effect on service user outcomes, therapeutic relationships, and clinical workflows. This will enable stakeholders to understand its utility, costs and requirements, informing its adoption in services for people with psychosis.

## Supplementary material

Supplementary material is available at https://academic.oup.com/schizophreniabulletin.

sbaf043_suppl_Supplementary_Material

## References

[1] Solmi M, Seitidis G, Mavridis D, et al Incidence, prevalence, and global burden of schizophrenia - data, with critical appraisal, from the Global Burden of Disease (GBD) 2019. Mol Psychiatry. 2023;28:5319–5327. https://doi.org/10.1038/s41380-023-02138-4 37500825

[2] World Health Organization. *World Mental Health Report.*; 2022. Accessed December 10, 2024. https://www.who.int/teams/mental-health-and-substance-use/world-mental-health-report

[3] Bhattacharyya S, Schoeler T, Di Forti M, Murray R, Cullen AE, Colizzi M. Stressful life events and relapse of psychosis: analysis of causal association in a 2-year prospective observational cohort of individuals with first-episode psychosis in the UK. Lancet Psychiatry. 2023;10:414–425. https://doi.org/10.1016/S2215-0366(23)00110-4 37146625 PMC10728826

[4] Munro J, Osborne S, Dearden L, Pascoe K, Gauthier A, Price M. Hospital treatment and management in relapse of schizophrenia in the UK: associated costs. Psychiatrist. 2011;35:95–100. https://doi.org/10.1192/pb.bp.109.027714

[5] Almond S, Knapp M, Francois C, Toumi M, Brugha T. Relapse in schizophrenia: costs, clinical outcomes and quality of life. Br J Psychiatry. 2004;184:346–351. https://doi.org/10.1192/bjp.184.4.346 15056580

[6] Ascher-Svanum H, Zhu B, Faries DE, et al The cost of relapse and the predictors of relapse in the treatment of schizophrenia. BMC Psychiatry. 2010;10:2. https://doi.org/10.1186/1471-244X-10-2 20059765 PMC2817695

[7] Fitzgerald P, De Castella A, Arya D, et al The Cost of Relapse in Schizophrenia and Schizoaffective Disorder. Australas Psychiatry. 2009;17:265–272. https://doi.org/10.1080/10398560903002998 19585288

[8] World Health Organization. *Mental Health Atlas 2020*.; 2021. Accessed December 10, 2024. https://www.who.int/publications/i/item/9789240036703

[9] MacDonald K, Fainman-Adelman N, Anderson KK, Iyer SN. Pathways to mental health services for young people: a systematic review. Soc Psychiatry Psychiatr Epidemiol. 2018;53:1005–1038. https://doi.org/10.1007/s00127-018-1578-y 30136192 PMC6182505

[10] World Health Organization. *Global Strategy on Digital Health 2020-2025.*; 2021. Accessed December 10, 2024. https://www.who.int/publications/i/item/9789240020924

[11] National Health Service. *Taskforce Mental Health. The Five Year Forward View For Mental Health.*; 2016. Accessed December 10, 2024. https://www.england.nhs.uk/wp-content/uploads/2016/02/Mental-Health-Taskforce-FYFV-final.pdf

[12] National Insitute for Health and Care Excellence. Evidence standards framework (ESF) for digital health technologies. Published online 2018. Accessed December 10, 2024. https://www.nice.org.uk/about/what-we-do/our-programmes/evidence-standards-framework-for-digital-health-technologies

[13] Gleeson JF, McGuckian TB, Fernandez DK, et al Systematic review of early warning signs of relapse and behavioural antecedents of symptom worsening in people living with schizophrenia spectrum disorders. Clin Psychol Rev. 2024;107:102357. https://doi.org/10.1016/j.cpr.2023.102357 38065010

[14] Gumley AI, Bradstreet S, Ainsworth J, et al The EMPOWER blended digital intervention for relapse prevention in schizophrenia: a feasibility cluster randomised controlled trial in Scotland and Australia. Lancet Psychiat. 2022;9:477–486. https://doi.org/10.1016/S2215-0366(22)00103-1 35569503

[15] Torous J, Bucci S, Bell IH, et al The growing field of digital psychiatry: current evidence and the future of apps, social media, chatbots, and virtual reality. World Psychiatry. 2021;20:318–335. https://doi.org/10.1002/wps.20883 34505369 PMC8429349

[16] Berardi C, Antonini M, Jordan Z, Wechtler H, Paolucci F, Hinwood M. Barriers and facilitators to the implementation of digital technologies in mental health systems: a qualitative systematic review to inform a policy framework. BMC Health Serv Res. 2024;24:243. https://doi.org/10.1186/s12913-023-10536-1 38408938 PMC10898174

[17] Bucci S, Schwannauer M, Berry N. The digital revolution and its impact on mental health care. Psychol Psychother. 2019;92:277–297. https://doi.org/10.1111/papt.12222 30924316

[18] Martinez-Martin N, Insel TR, Dagum P, Greely HT, Cho MK. Data mining for health: staking out the ethical territory of digital phenotyping. NPJ Digital Med. 2018;1:68. https://doi.org/10.1038/s41746-018-0075-8 PMC6550156 31211249

[19] Barnett I, Torous J, Staples P, Sandoval L, Keshavan M, Onnela JP. Relapse prediction in schizophrenia through digital phenotyping: a pilot study. Neuropsychopharmacology 2018;43:1660–1666. https://doi.org/10.1038/s41386-018-0030-z 29511333 PMC6006347

[20] Ben-Zeev D, Brian R, Wang R, et al CrossCheck: Integrating self-report, behavioral sensing, and smartphone use to identify digital indicators of psychotic relapse. Psychiatr Rehabil J. 2017;40:266–275. https://doi.org/10.1037/prj0000243 28368138 PMC5593755

[21] Smith KA, Blease C, Faurholt-Jepsen M, et al Digital mental health: challenges and next steps. BMJ Ment Health. 2023;26:e300670. https://doi.org/10.1136/bmjment-2023-300670 PMC10231442 37197797

[22] Kickbusch I, Piselli D, Agrawal A, et al; Secretariat of the Lancet and Financial Times Commission. The Lancet and Financial Times Commission on governing health futures 2030: growing up in a digital world. Lancet (London, England). 2021;398:1727–1776. https://doi.org/10.1016/S0140-6736(21)01824-9 34706260

[23] Mohr DC, Lyon AR, Lattie EG, Reddy M, Schueller SM. Accelerating digital mental health research from early design and creation to successful implementation and sustainment. J Med Internet Res. 2017;19:e153. https://doi.org/10.2196/jmir.7725 28490417 PMC5443926

[24] Camacho E, Torous J. Interest and readiness for digital mental health in coordinate specialty care for early course psychosis: A survey study of 42 programs in 30 states. Early Interv Psychiatry. 2021;15:1243–1255. 33260266 10.1111/eip.13073

[25] Dominiak M, Gędek A, Antosik AZ, Mierzejewski P. Mobile health for mental health support: a survey of attitudes and concerns among mental health professionals in Poland over the period 2020-2023. Front Psychiatry. 2024;15:1303878. 38559395 10.3389/fpsyt.2024.1303878 PMC10978719

[26] Miller MJ, Eberhart LG, Heliste JL, Tripuraneni BR. Patient and health care professional perspectives about referral, self-reported use, and perceived importance of digital mental health app attributes in a diverse integrated health system: cross-sectional survey study. JMIR Formative Research. 2024;8:e59831. 39546791 10.2196/59831 PMC11607546

[27] Bucci S, Berry N, Morris R, et al “They are not hard-to-reach clients. We have just got hard-to-reach services.” Staff views of digital health tools in specialist mental health services. Front Psychiatry. 2019;10:344. 31133906 10.3389/fpsyt.2019.00344 PMC6524662

[28] Allan S, Bradstreet S, Mcleod H, et al; EMPOWER Group. Developing a hypothetical implementation framework of expectations for monitoring early signs of psychosis relapse using a mobile app: qualitative study. J Med Internet Res. 2019;21:e14366. https://doi.org/10.2196/14366 31651400 PMC6838692

[29] Byrne S, Tohamy A, Kotze B, et al Using a mobile health device to monitor physiological stress for serious mental illness: A qualitative analysis of patient and clinician-related acceptability. Psychiatr Rehabil J. 2022;45:219–225. 35298226 10.1037/prj0000514

[30] Stefancic A, Rogers RT, Styke S, et al Development of the first episode digital monitoring mhealth intervention for people with early psychosis: qualitative interview study with clinicians. JMIR Mental Health. 2022;9:e41482. 36331539 10.2196/41482 PMC9675009

[31] Rogan J, Bucci S, Firth J. Health care professionals’ views on the use of passive sensing, ai, and machine learning in mental health care: systematic review with meta-synthesis. JMIR Ment Health. 2024;11:e49577. https://doi.org/10.2196/49577 38261403 PMC10848143

[32] Fletcher AJ. Applying critical realism in qualitative research: methodology meets method. Int J Soc Res Methodol. 2017;20:181–194. https://doi.org/10.1080/13645579.2016.1144401

[33] Tong A, Sainsbury P, Craig J. Consolidated criteria for reporting qualitative research (COREQ): a 32-item checklist for interviews and focus groups. Int J Qual Health Care. 2007;19:349–357. https://doi.org/10.1093/intqhc/mzm042 17872937

[34] Douglas H. Sampling techniques for qualitative research. In: Islam MR, Khan NA, Baikady R, eds. Principles of Social Research Methodology. Springer Nature Singapore; 2022:415–426. https://doi.org/10.1007/978-981-19-5441-2_29

[35] Braun V, Clarke V. Thematic Analysis: A Practical Guide. SAGE; 2022.

[36] NVivo. Published online 2018.

[37] Braun V, Clarke V. Toward good practice in thematic analysis: Avoiding common problems and be(com)ing a knowing researcher. Int J Transgend Health. 2023;24:1–6. https://doi.org/10.1080/26895269.2022.2129597 36713144 PMC9879167

[38] Mental Health Act. UK Government; 1983. https://www.legislation.gov.uk/ukpga/1983/20/contents

[39] Eisner E, Drake RJ, Berry N, et al Development and long-term acceptability of ExPRESS, a mobile phone app to monitor basic symptoms and early signs of psychosis relapse. JMIR Mhealth Uhealth. 2019;7:e11568. https://doi.org/10.2196/11568 30924789 PMC6460313

[40] Bucci S, Morris R, Berry K, et al Early psychosis service user views on digital technology: qualitative analysis. JMIR Ment Health. 2018;5:e10091. https://doi.org/10.2196/10091 30381280 PMC6236205

[41] Lobban F, Appelbe D, Appleton V, et al IMPlementation of An online Relatives’ Toolkit for psychosis or bipolar (IMPART study): iterative multiple case study to identify key factors impacting on staff uptake and use. BMC Health Serv Res. 2020;20:219. https://doi.org/10.1186/s12913-020-5002-4 32183787 PMC7077000

[42] Bell IH, Lim MH, Thomas N. The therapeutic use of digital technologies in psychosis. In: A Clinical Introduction to Psychosis. Elsevier; 2020:637–662. https://doi.org/10.1016/B978-0-12-815012-2.00027-4

[43] Berry N, Bucci S, Lobban F. Use of the internet and mobile phones for self-management of severe mental health problems: qualitative study of staff views. JMIR Ment Health. 2017;4:e52. https://doi.org/10.2196/mental.8311 29092809 PMC5688247

[44] Aref-Adib G, McCloud T, Ross J, et al Factors affecting implementation of digital health interventions for people with psychosis or bipolar disorder, and their family and friends: a systematic review. The Lancet Psychiatry. 2019;6:257–266. https://doi.org/10.1016/S2215-0366(18)30302-X 30522979

[45] Eisner E, Faulkner S, Allan S, et al Barriers and facilitators of user engagement with digital mental health interventions for people with psychosis or bipolar disorder: systematic review and best-fit framework synthesis. JMIR Mental Health. Published online (in press);12(1):e65246. 39832352 10.2196/65246 PMC11791459

[46] Brown P, Waite F, Lambe S, et al Automated virtual reality cognitive therapy (gameChange) in inpatient psychiatric wards: qualitative study of staff and patient views using an implementation framework. JMIR Formative Research. 2022;6:e34225. 35412462 10.2196/34225 PMC9044147

[47] Tauginienė L, Hummer P, Albert A, Cigarini A, Vohland K. Ethical challenges and dynamic informed consent. In: Vohland K, Land-Zandstra A, Ceccaroni L, et al, eds. The Science of Citizen Science. Springer International Publishing; 2021:397–416. https://doi.org/10.1007/978-3-030-58278-4_20

[48] Slade M, Amering M, Farkas M, et al Uses and abuses of recovery: implementing recovery-oriented practices in mental health systems. World Psychiatry. 2014;13:12–20. https://doi.org/10.1002/wps.20084 24497237 PMC3918008

[49] Saeb S, Lattie EG, Schueller SM, Kording KP, Mohr DC. The relationship between mobile phone location sensor data and depressive symptom severity. PeerJ. 2016;4:e2537. https://doi.org/10.7717/peerj.2537 28344895 PMC5361882

[50] Nicholas J, Shilton K, Schueller SM, Gray EL, Kwasny MJ, Mohr DC. The role of data type and recipient in individuals’ perspectives on sharing passively collected smartphone data for mental health: cross-sectional questionnaire study. JMIR Mhealth Uhealth. 2019;7:e12578. https://doi.org/10.2196/12578 30950799 PMC6473465

[51] Mehta N. Mind-body dualism: A critique from a health perspectiveFNx08. Mens Sana Monogr. 2011;9:202–209. https://doi.org/10.4103/0973-1229.77436 21694971 PMC3115289

[52] Eisner E, Drake R, Lobban F, Bucci S, Emsley R, Barrowclough C. Comparing early signs and basic symptoms as methods for predicting psychotic relapse in clinical practice. Schizophr Res. 2018;192:124–130. https://doi.org/10.1016/j.schres.2017.04.050 28499766 PMC5821684

[53] Granja C, Janssen W, Johansen MA. Factors determining the success and failure of ehealth interventions: systematic review of the literature. J Med Internet Res. 2018;20:e10235. https://doi.org/10.2196/10235 29716883 PMC5954232

[54] D’Alfonso S, Coghlan S, Schmidt S, Mangelsdorf S. Ethical dimensions of digital phenotyping within the context of mental healthcare. J Technol Behav Sci. 2024;0. https://doi.org/10.1007/s41347-024-00423-9

[55] Fricker M. Epistemic Injustice. Oxford University Press; 2007. https://doi.org/10.1093/acprof:oso/9780198237907.001.0001

[56] Speyer H, Eplov LF, Roe D. Antipsychotic Discontinuation through the Lens of Epistemic Injustice. Community Ment Health J. 2024;61:244–247. https://doi.org/10.1007/s10597-024-01274-7 38587713 PMC11772521

[57] Schmidt S, D’Alfonso S. Clinician perspectives on how digital phenotyping can inform client treatment. Acta Psychol (Amst). 2023;235:103886. https://doi.org/10.1016/j.actpsy.2023.103886 36921359

[58] Williams A, Farhall J, Fossey E, Thomas N. Internet-based interventions to support recovery and self-management: a scoping review of their use by mental health service users and providers together. BMC Psychiatry. 2019;19:191. https://doi.org/10.1186/s12888-019-2153-0 31221125 PMC6585058

