# Supplementary material for: Mental Health Professionals’ Perspectives on Digital Remote Monitoring in Services for People with Psychosis
Source: Schizophr Bull. 2025 May 7;52(1):sbaf043. doi: 10.1093/schbul/sbaf043 (PMC12809784; doi:10.1093/schbul/sbaf043)
Supplement: sbaf043_suppl_Supplementary_Material [file sbaf043_suppl_supplementary_material.zip › Supplementary material_Interview Topic Guide.docx]

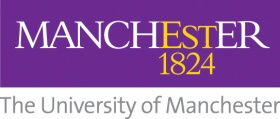
Topic Guide (Staff)

**Study title:** A qualitative study of service user and staff views on digital remote monitoring for unusual distressing experiences (psychosis)

| **Participant ID number** |  |
| --- | --- |
| **Notes can be made on this topic guide. However, please do not include identifiable information and do dispose of any paper copies securely once the interview has finished.**  **When you finish the interview, you will need to complete a reflective log via Qualtrics** [**https://www.qualtrics.manchester.ac.uk/jfe/form/SV_ddqP2eGP0wFY97M**](https://www.qualtrics.manchester.ac.uk/jfe/form/SV_ddqP2eGP0wFY97M)  **Please ensure that this log is completed after each participant and that actions based on the log are taken to the project management group as soon as possible.** | |
| **Prior to interview, research workers should:**  Send participant the consent form (in their preferred format) and participant information sheet.  Check they have arranged a time to meet/speak with the participant and, if applicable, that a Zoom (or similar) online meeting invite has been sent to the participant and that they have accepted.  The Research Assistant and participant should sit as close to the microphone as possible when using a dictaphone to ensure everything can be easily heard on the recording | |

**Introductions**

- My name is [researcher name]. I’m a researcher on the CONNECT study which is a study about using smartphones and wearable devices (like Fitbits, step trackers or smart watches) to help people keep track of their mental health.
- Thank you for agreeing to meet with me today for the research interview and for reading the participant information sheet and filling in the consent form. Before we get started, do you have questions about anything in the participant information sheet or the consent form? [Answer any questions].

Re-confirm informed consent is still valid and participant still wishes to take part. Key points to cover:

- Interview length: The interview today will last up to 60 minutes.
- Audio recording: We will audio record the interview.
- Anonymity: We will store the interview recording and all other study data securely using an anonymous number rather than your name. It’s best to avoid using full names in the interview, but all names will be removed when the interview is transcribed. We might use quotes from the interview when we publish the research, but the quotes will not allow you to be identified.
- Limits of confidentiality: What you say in the interview will remain confidential unless something you say makes me concerned that you or someone else might come to some harm. In that case, I would have to pass the information on to another relevant person.
- Voluntary. It’s your choice to decide whether or not to take part. You can take a break or stop the interview completely at any point without giving a reason (and without penalty).

Are you still happy to take part in the interview?

*[If yes]* Ok, thanks, I’m going to start the recording now ***[Start audio recording]***

Note to research workers: **What follows is a guide.** The order and exact content of the questions will be determined by the participant and will be influenced by the ongoing analysis, so the order of the questions may vary as the interview develops. Prompt and ask for examples as time permits.

Explanations of key concepts and the main interview questions are in blue. Follow-up questions to prompt further discussion are included *in italics*.

| **Topic** | **Interview script/questions/prompts** | **Notes** |
| --- | --- | --- |
| **Overview** | |  |
| Overview of interview | In this interview, I’d like to explore what you think about how smartphones, wearable devices and machine learning can be used to help manage service users’ mental health. Don’t worry if these terms aren’t quite clear yet – we will explain them during the interview. I’d like to understand what you think about this topic in general… and I’m also really keen to hear your views about some particular ways that these devices and methods can be used with service users and what impact this has on clinical workflows.  I’ll start out with some general questions. Then I’ll describe those particular scenarios in more detail and ask what you think about them. Does that sound ok? |  |
| Warm up Qs to set the scene and help the researcher know how to pitch later questions | To start with, for context, please can you tell me a little bit about your own current use of digital devices, such as smartphones, wearable devices and so on?  Do you personally use a smartphone or a wearable device like a smartwatch, Fitbit or step tracker at the moment?  [If yes] What do you tend to use it/them for? *How often? (Daily? Less?)*  What is your general view about smartphones and wearable devices like Fitbits, step-trackers or smartwatches?  What do you think of the idea of using devices like smartphones or wearables for health purposes? That could be for physical health, fitness, or mental health… | If the participant mentions a specific wearable, you can use that specific example throughout the interview, as applicable. If they use more than one device, make sure you ask questions about each device, as applicable |
| What are the barriers/facilitators to service users using digital tools for healthcare? | What experiences have you had in using digital health tools like apps, websites, fitness trackers, etc as part of your clinical work with service users?  *Probe: If yes to experience, Did it work well? Did it not work well?*  *Probe: If no experience, Are there any reasons why you have not used digital tools as part of your delivery of clinical care to service users?*  What do you think about service users using technology like this to help keep track of health?  How are digital health tools (apps, fitness trackers, websites) currently used in your service?  *Probe: If used in service, Did it work well? Did it not work well?*  *Probe: If not used in service, Are there are any reasons why you have not used digital tools as part of your delivery of clinical care to service users?* |  |
| **Symptom monitoring** | |  |
| Description of symptom monitoring | One way smartphones can be used is to keep track of a service user’s symptoms or how they have been feeling day-to-day.  I’ve got an example of an app that helps people do this. It’s called the ClinTouch app. [Show Figure 2]  How this works is the app prompts the person each day to answer a few questions about their mood and experiences (like whether a person has been feeling low, and whether they have been bothered by voices). It can also ask about where they are and who they are with, to check out whether certain situations, places or people change how a person might be feeling.  The app can then send the responses electronically to the individual’s mental health team so they know if the service user needs any extra support, like an extra visit or a doctor’s appointment. | Figure 1: Example symptom monitoring app question (ClinTouch app) |
| What are staff’s concerns about active symptom monitoring and what do they perceive the benefits to be? | How would you feel about asking service users on your caseload to use an app to help you both keep track of their mental health in this way?  *What would you as a clinician find helpful about a service user keeping track of their symptoms or feelings in this way? Are there any benefits?*  *Would anything worry you about asking a service user to use an app to keep track of their symptoms or feelings in this way? [If yes, what in particular?]* |  |
| How can we keep service users engaged with symptom monitoring? | What do you think about service users tracking mental health in this way long term?  *For example, in our CONNECT research study, we will ask people to track their mental health for 12 months. Do you think service users would track their mental health for this long? Why? Why not?*  What would help service users keep on tracking their mental health like this long term?  *What could help motivate them? Is there anything we could build into the app to help?* |  |
| **Passive sensing** | |  |
| Description of passive sensing | Smartphones and wearables (like Fitbits or smartwatches) can gather lots of continuous information, without a person having to *do* anything apart from wear the wearable or carry their phone around with you. This is called *passive sensing.* Here are some examples [show Figure 2 and give the participant some time to look at the examples of info tech can gather].  This information tells a story about the kind of things someone has been doing – like whether they’ve been sleeping, exercising, travelling, or phoning people.  In turn, this can give clues about how a person might be feeling. I’ll give you an example of how this could work:   - Someone who is feeling very anxious or low might not feel like socialising much. - They might stay at home more than usual and phone their friends less. - Because their smartphone can gather information about where they’ve been and how many phone calls they’ve made, it could quickly spot that change. - Allowing their phone to spot changes like this might help the person know that they might need some extra support. This information could be sent to their mental health team too, so they could quickly offer that support. This could help keep them safe and well.   What it can’t tell us, though, is why someone might be staying at home more than usual, or why someone’s sleep might be affected.  Do you have any questions about how passive sensing works or anything I have just said? [clarify any uncertainty] | Figure 2: Types of information gathered by smartphones and wearable devices |
| What are staff member’s concerns about passive sensing and what do they perceive the benefits/challenges to be? | What do you think about service users collecting health-related information in this way?  *How do you feel about this? Would you feel comfortable with service users on your caseload collecting information in this way? [If yes/no] What in particular might you feel comfortable or uncomfortable about?*  *What do you think about this passive sensing technology being used to monitor symptoms of psychosis specifically?*  *- How might it benefit service users? Do you have any concerns?*  *- How do you think service users will find this? (Anticipated problems/benefits - how might it help the service user? What might the benefits be?)*  *How might it help you as a clinician or the clinical team in terms of help to support / manage a service user’s mental health / care? What might the benefits be? What are the challenges?*  *What do you think about asking service users to collect information in this way long term? (e.g. we will ask people to do so for 12 months in our Connect research study)*  *Have you come across/recommended anything like this to service users in your clinical practice?*  *- How do you find doing this?*  *- What has the feedback been like?*  *- How does it support your practice/intervention?* | Note: If the staff member seems unsure or say they are not sure, clarify what the specific uncertainty is and whether they need more explanation about what passive sensing does and how it works |
| What can be done to make passive sensing technology more acceptable to users (including both service users and the clinical team)? | [If they have concerns…] What would help you as a clinician feel more comfortable about health-related information being gathered in this way?  What would help the clinical team feel more comfortable about health-related information being gathered in this way? What would the impact be on the clinical team?  *How can we make it as easy and acceptable as possible for users (including both service user and clinical management)?*  *What do you think we could do to reassure you about gathering this type of health-related information from smartphones or wearables (including both service user and clinical management)?*  *Would you want to see a copy of the information the smartphone or wearable has gathered? [If yes] How would you want to receive this? (e.g. in the app, website). How often would you want to see this? (E.g. once a week, once a month?).*  Smartphones can gather information about where people are throughout the day.   - How would you feel about the app or potentially the clinical team knowing a service user’s exact location? - How would you feel about the app or potentially the clinical team knowing the general area that a service user was in (e.g. [insert relevant local area. E.g. Fallowfield])? - How would you feel about the app or potentially the clinical team knowing how far a service user has travelled from home each day (without knowing where the service user was travelling exactly to or from)? - *Which of these options would you feel most comfortable with?* |  |
| **Relapse** | |  |
| Description of relapse | In the CONNECT study, we want to help people to manage their mental health and minimise relapses. One way of doing this is to spot early when people are starting to relapse. Today, we’ve talked about ways that technology could help people keep track of their mental health – by allowing them to report their symptoms in an app and by gathering ongoing health-related information from the smartphone or wearable. In our CONNECT research study, we are looking at whether we can use these methods to spot early signs of relapse. |  |
| What are staff member’s views on active/passive monitoring for *relapse prediction* specifically? | What do you think about using these methods to spot early signs that a service user might be experiencing an impending psychosis relapse?  *What would you consider the benefits of using technology to spot relapse early?*  *Would anything worry you about using technology to do this?*  What do you see your role to be in this?  What implications might this have on routine clinical practice/clinical management?  *Which service users might benefit from a digital remote monitoring system like this? Why is this? Who would not benefit? Why?* |  |
| **Machine learning** | |  |
| Description of machine learning | Using smartphones and wearables in the ways we’ve talked about collects a large amount of health-related information. For that information to be useful, we need a way of analysing it. One way of making sense of it is to use a technique called **machine learning.**  YouTube and Netflix use this technique. They gather lots of information about people’s viewing patterns and then make recommendations to viewers about what they might want to watch next.  In our CONNECT research study, we’ll use smartphones and wearables to collect a lot of health-related information from a lot of people with psychosis. Then we’ll see if we can use machine learning methods to detect a relapse.   - To do this, first we’ll have a training phase where we teach a computer to recognise patterns in the information we’ve collected. For example, one pattern might be that people tend to leave the house less as their mental health is worsening. - During this training phase, while the computer is learning to spot patterns, it gets lots of human guidance and supervision. - When the computer has finished its training, it can then spot the patterns it’s learnt without needing human supervision. So it could spot that someone’s mental health may be worsening and let their care team know.   This process is a bit like when an athlete is training for a big race. First they’ll spend a lot of time training, with their coach always there with them – supervising them and pointing out how they can improve. Then once the training is finished, the athlete has learnt all that they need to go ahead and do the race on their own, without the coach. |  |
| What are staff members’ views on machine learning for *relapse prediction* specifically? | How do you feel about this process of a computer program learning to pick up or detect a possible relapse?  *Do you have any comments or concerns about this method? [If yes: how so? Can you tell me a bit more about that? What are your concerns?]*  *Is there anything that would worry you about a computer program picking up when a service user might be getting unwell? [If yes, what in particular?]* |  |
| What concerns do staff members have around machine learning systems/ algorithms? | *What would you need to know to feel comfortable about your service using this method as part of routine healthcare?*  *What would we need to consider, from your point of view, to make this an acceptable way for services to support service users? What would reassure you?*  *Is there anything we could do to put your mind at ease about using this method?* |  |
| What proportion of false negatives and false positives is acceptable for predicting relapse? | People get things wrong sometimes… as you know, you can’t *always* predict when a service user might be experiencing a relapse. You might think a service user is about to relapse when they don’t, and other times you might miss a relapse.  Just like clinicians can get things wrong sometimes, so can computer programs. The program won’t *always* get it right. The computer might think an individual’s mental health is getting worse when actually it isn’t… and it might sometimes miss when a service user is becoming unwell.  What do you think about the idea that the program won’t *always* predict an impending relapse?  *Does this matter a great deal to you? [If so, why?]*  *What concerns might you have about this?*  *Would this affect your willingness to use a machine learning system that aims to predict relapse?*  *As a clinician, which would be worse for you – the computer says a service user is about to relapse when they aren’t… or if the computer did not pick up a relapse when a service user was about to relapse (i.e. it got missed)? Why is that?* |  |
| **Sharing data with mental health team and others** | |  |
|  | How would you feel about an app alerting you / the clinical service that a service user may be relapsing?  *How would you feel about having to see health-related information collected from a service user’s smartphone or wearable? What particular concerns might you have?*  *Are there reasons why seeing data collected in this way would be helpful?*  *What would help you feel comfortable with this way of working?*  *In general, how would you want this to work for you? (e.g. in app alert, email, text)*  *What support do you think you or other clinicians might need to use a digital remote monitoring system described?* |  |
|  | In future, a system that uses smartphones or wearable devices to help people keep track of their mental health might eventually become part of the standard care offered by mental health teams. I’m interested to hear your views on how this might work.  One scenario might be that everyone under the care of a mental health team would need to agree to use a smartphone/wearable system as a condition of being offered mental healthcare. What is your view on this?  Should people be able to opt out of using a system like this? Why? | [Note: this is intentionally controversial to get participants thinking about what they would or would not be happy with] |
| **Integration of digital remote monitoring system into clinical practice** | | |
|  | Where do you think a digital remote monitoring system like this would fit in the context of supporting service users with psychosis?  Where do you think it would fit to support you in providing the best care to a service user? How might a digital remote monitoring system be used in your service?  *Is there anything that would get in the way of you using this type of system?*  *What impact would a system like this have on your workload / practice?*  *What do you foresee would facilitate or act as barriers to continued engagement with a digital remote system like CONNECT?*  *Probe: What would make it more difficult / harder for service users to remain engaged? What would make it more difficult / harder for you to remain engaged with this system?*  What support would you/staff need to use a digital remote monitoring system like CONNECT? (Training? Extra time? Ongoing support from a digital champion?) |  |
| **CONNECT** | |  |
| Views on ethical/ governance issues associated with data tracking/storage in a digital health context | What could we do to help you trust this kind of system?  What do you think we could do to help you feel confident that the information we collect would be stored safely and securely?  *Who should have access the smartphone or wearable information?* |  |

**Appendix A: Service Managers / Team Leads**

Introduction:

In this interview, I’d like to explore what you think about using digital tools to help manage service users’ mental health. I will start by briefly explaining the CONNECT study and then move on to asking some questions. Does that sound OK?

Description of CONNECT:

As you know, psychosis runs a relapsing course - most people will experience a relapse within 5 years of a first episode of psychosis. We know that accurately predicting when a service user might relapse is a challenge. In CONNECT, we are looking to see if we can predict relapse in advance of it happening.

We will be collecting three types of data over 12-months to do this. Through the CONNECT app, participants will be asked to respond to a set of questions about their symptoms and mental health several times a week. We will also collect information about people’s sleep and activity levels, for example, from sensors on a smartphone that we will ask people to carry around with them and a wrist-worn wearable device like a Fitbit or Apple watch. We will also meet with the participants every 3 months and support them in completing some questionnaires about their health and mental health.

At the end of the study, after we have collected this information, we will separate the data into those who experienced a relapse over the 12-month period and those who did not. We will then look back and see if we can find patterns (a bit like early warning signs) in the data that show a change in behaviour leading up to a relapse. For example, we might find that changes in sleep patterns, reduced physical activity and not getting out and about were the things that predicted when more people relapsed compared to those who did not. In the future, a digital remote monitoring system like could be used to spot early that someone’s mental health is worsening and then notify services so that their care team can offer timely support.

1. What do you think about the idea of using digital tools such as these being offered/delivered in services?
2. Who do you think might benefit from using digital tools like these? (and why / why not)
3. What do you think are the advantages of digital tools such as these being offered / delivered…
   1. For the service user?
   2. For professionals?
   3. For the service?
4. What do you think are the disadvantages of digital tools such as these being offered / delivered…
   1. For the service user?
   2. For professionals?
   3. For the service?
5. At what point in the care pathway would digital tools fit to support you in providing the best care to service users? How might a digital remote monitoring system that is used to pick up service users’ early warning signs be used in your service(s)?
6. Do you feel that using digital tools to monitor service user’s symptoms or to provide interventions and support is the right way to support service users with psychosis?

Prompt: *Do you have any concerns with this population using these tools / approaches?*

*Do you think there is a better way?*

1. If you decided to support a study like the CONNECT study (explain the study again if needed), what do you hope it would achieve for your service/service users/clinicians?
2. What is currently offered in your service(s) to monitor early warning signs and predict relapse? How does what is already offered function in your service already?

In what ways do you think the digital tools we have talked about today compares to this / differs from the usual ways you would support service users? / How do they fit with what is already offered? How would it be different?

1. What would using a digital remote monitoring system like this mean from a service organisation and delivery point of view? What work would we need to do to embed this in services? How could we ensure it is used and becomes part of routine service?
2. What do you foresee would facilitate / help uptake of a system like this being offered / delivered (could ask again in terms of the different levels – service user, professionals, service) within routine practice?
   1. What do you think would be the most important things for getting digital tools like those we have discussed today rolling in your service / services in future?
   2. Are there any reasons why using digital tools to support people with psychosis might be attractive to the NHS?
   3. Who would we need to get on board to make digital tools a viable part of service delivery?
3. Do you have any reservations for either the service user group (e.g. risk concerns) or for the team (e.g. safeguarding, workloads, admin)?
   1. Prompt: *What do you think would be the reservations amongst staff / any difficulties in establishing buy in?*
4. What barriers do you foresee would impede uptake of a system like this? (example probe – practicalities) within routine practice?
5. What do you foresee would facilitate / help continued engagement with this digital system?
6. What is the impact of introducing new innovations in healthcare like digital tools on you as a service manager?
7. What have you scaled up before? How have you gone about doing this in the past? (this will provide an insight into how the service will approach things in the future)
   1. What do you think it would be important for service managers to do to implement digital tools into clinical service pathways?
   2. Would anything need to change in services for their implementation to be effective?
   3. How do you think we could help to establish buy in for using digital tools amongst managers/ service managers like yourself?
   4. What do you think the impact would be on resourcing?
   5. Would it lessen or increase workloads?
8. What are your thoughts about if and how this digital remote system could be scaled up and integrated within multiple services?

Interview closedown:

- Is there anything else that you would like to tell me that we haven’t discussed?
- How have you found this interview today?
- How do you think this interview could be improved for future participants?
- Ok, I’ll now switch off the audio recorder *[turn off recording]*

Thank you for taking part in the interview. Just before we finish, I’ll go through some very quick demographic questions with you. [Go through demographics Qs and input directly into Qualtrics - <https://www.qualtrics.manchester.ac.uk/jfe/form/SV_3DaiXFGw7oZiEDA> ]

Finishing:

- Thank participant for taking part and let them know that their contribution to this research is extremely useful and important.
- Explain what will happen now and how this information will be used
- Offer to provide summary of the findings when available

[Complete reflective log via Qualtrics - <https://www.qualtrics.manchester.ac.uk/jfe/form/SV_ddqP2eGP0wFY97M> ]

***Figure 1: Example symptom monitoring app question (ClinTouch app)***


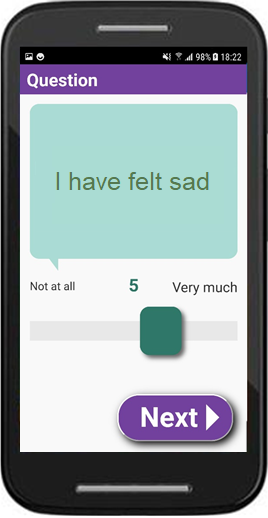


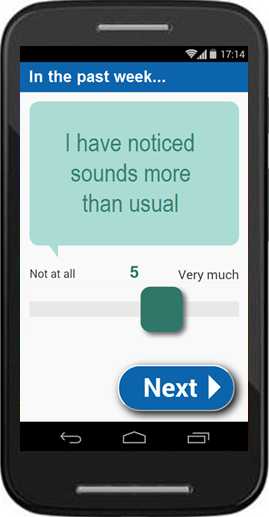

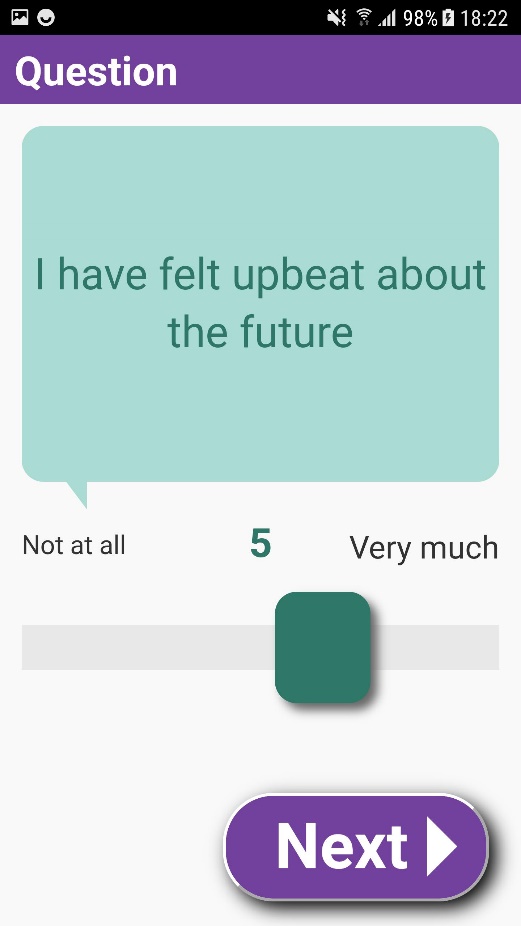

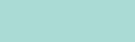


I have heard voices

***Figure 2: Types of information that can be gathered by smartphones and wearable devices***

Air temperature

Heart rate

Nearby Bluetooth devices


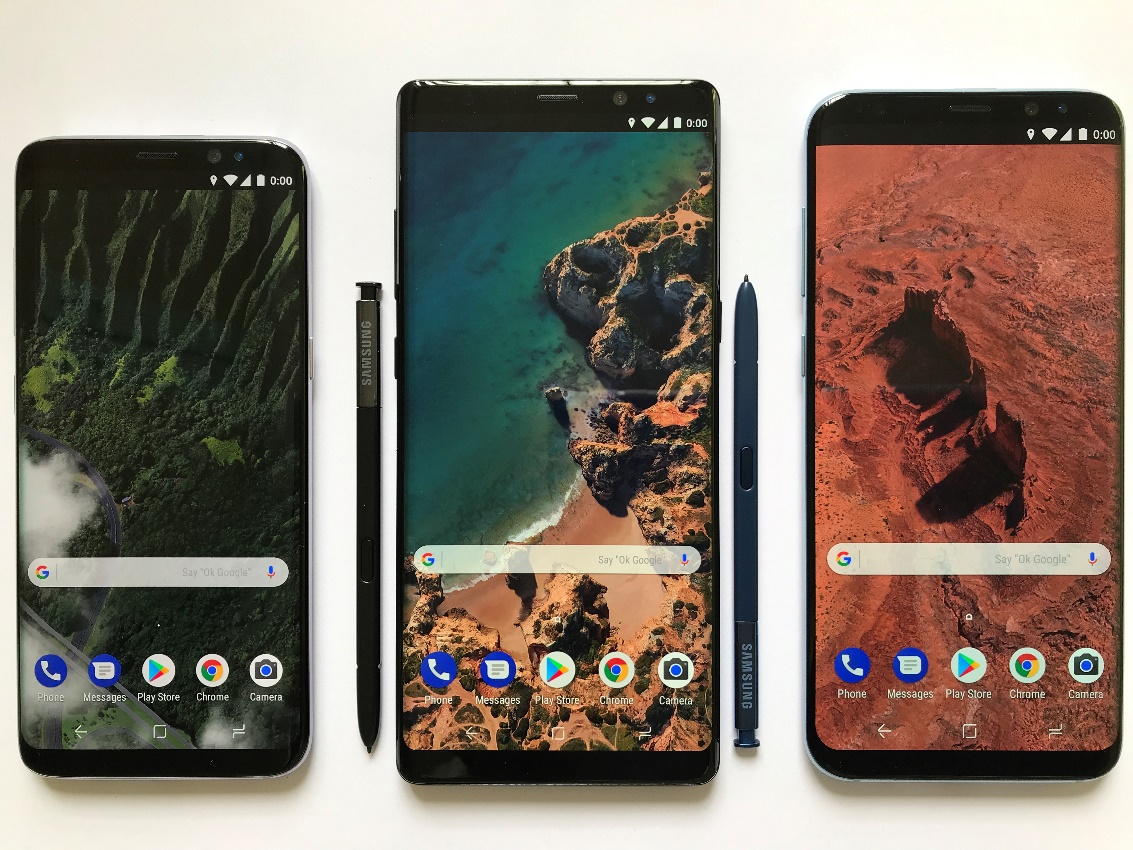


Skin temperature

Light levels

Step count

Number/length of phone calls


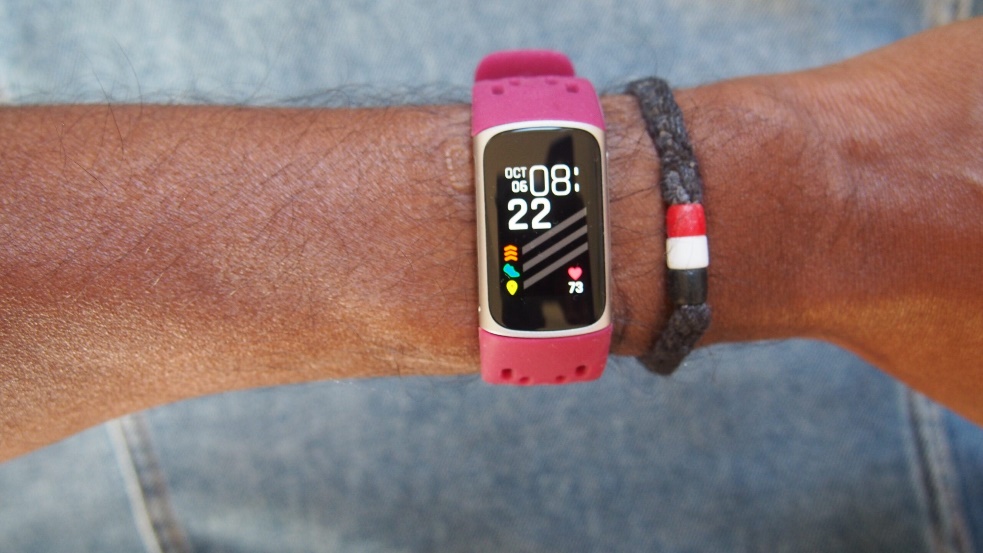


Minutes active

Number of text messages (sent/received)

Distance travelled

Calories used up

Location

App use

Sleep

**Information gathered by smartphones**

**Information gathered by wearables**
