# Supplementary material for: Mental Health Professionals’ Perspectives on Digital Remote Monitoring in Services for People with Psychosis
Source: Schizophr Bull. 2025 May 7;52(1):sbaf043. doi: 10.1093/schbul/sbaf043 (PMC12809784; doi:10.1093/schbul/sbaf043)
Supplement: sbaf043_suppl_Supplementary_Material [file sbaf043_suppl_supplementary_material.zip › Supplementary material_Reflexivity.docx]

**Reflexivity**  
 
All researchers were part of the CONNECT study research team which included academics, researchers, clinicians and people with lived experience of psychosis. Researchers who completed the interviews were primarily employed as local site researchers on the study. Interviewers (n=16) received training by SB, HB, and EE, who are experienced in qualitative research methodology. Training covered general background to qualitative research, interviewing skills and role-play interviews with feedback. HB, a female Clinical Psychologist and PhD researcher investigating the implementation of digital technologies in psychosis care, provided supervision to interviewers, which included listening to interview recordings and providing feedback. Most participants did not have pre-existing relationships with interviewers, but a small minority knew their interviewers in a professional capacity. All participants were aware that researchers were involved in the CONNECT study and thus may have assumed an inherent interest and favourable bias towards using digital technology in mental health care.
